# Ophthalmoparesis, papillitis and premacular hemorrhage in a case with endocarditis: A rare presentation of brucellosis

**DOI:** 10.4103/0301-4738.60098

**Published:** 2010

**Authors:** Özlem Gürses Sahin, Aysel Pelit, Tuğba Turunc, Yonca Aydin Akova

**Affiliations:** Department of Ophthalmology, Middle East Technical University, Cankaya, Ankara-064 50, Turkey; 1 Department of Ophthalmology, Baskent University Medical Faculty Hayri Cecen Sok. 29/10 Ataturk Sitesi ORAN, Cankaya, Ankara-064 50, Turkey; 2 Department of Infectious Diseases, Baskent University Medical Faculty Hayri Cecen Sok. 29/10 Ataturk Sitesi ORAN, Cankaya, Ankara-064 50, Turkey

**Keywords:** Brucellosis, endocarditis, retinal hemorrhage

## Abstract

We report a rare presentation of brucellosis as bilateral optic nerve and right abducent nerve involvement, and endocarditis complicated by right premacular hemorrhage in a 28-year-old white female. The patient showed improvement with both medical and surgical therapy. Brucellosis should be considered in the differential diagnosis of papillitis, gaze palsy and endocarditis complicated with premacular hemorrhage in endemic regions.

Brucellosis, which is endemic in Turkey, is a systemic infection that can affect any organ or system in the body.[1] The most common ocular manifestations of brucellosis are considered as anterior uveitis and choroiditis.[2] We report a rare case of brucellosis with bilateral involvement of optic nerve, involvement of right abducent nerve, and endocarditis complicated by premacular hemorrhage. To our knowledge, premacular hemorrhage secondary to brucella endocarditis is the first to be reported.

## Case Report

**Figure 1:**
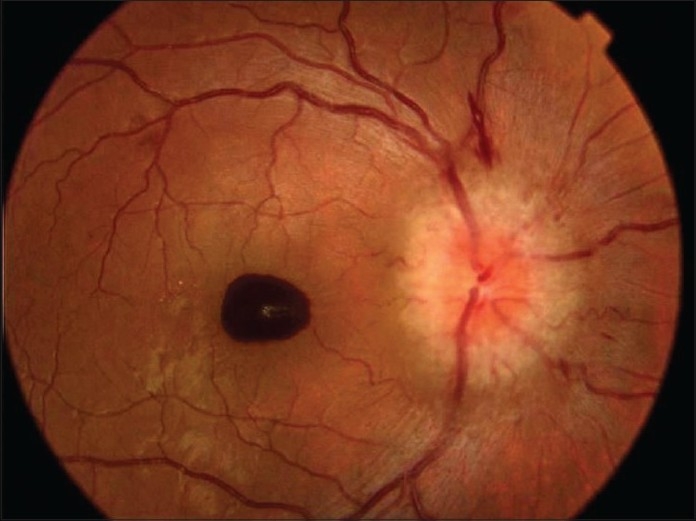
Color photo of the right eye at presentation. Note 1½ disc diameter of premacular hemorrhage, marked optic disc swelling and hyperemia, peripapillary vascular changes and flame-shaped hemorrhages

**Figure 2:**
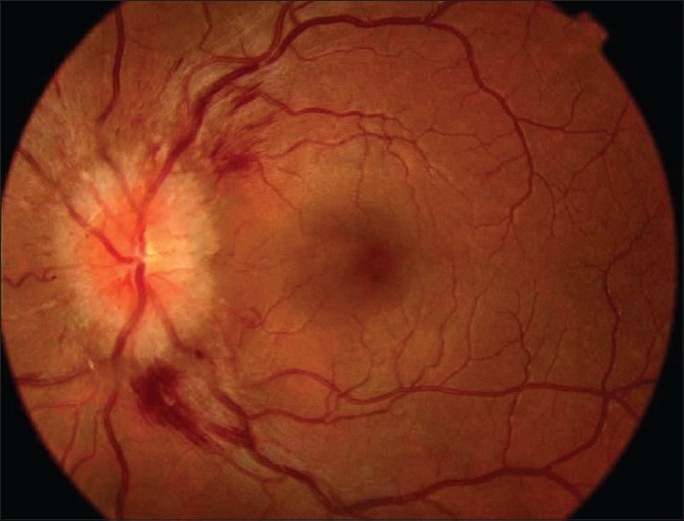
Color photo of the left eye at presentation. Note marked optic disc swelling and hyperemia

**Figure 3:**
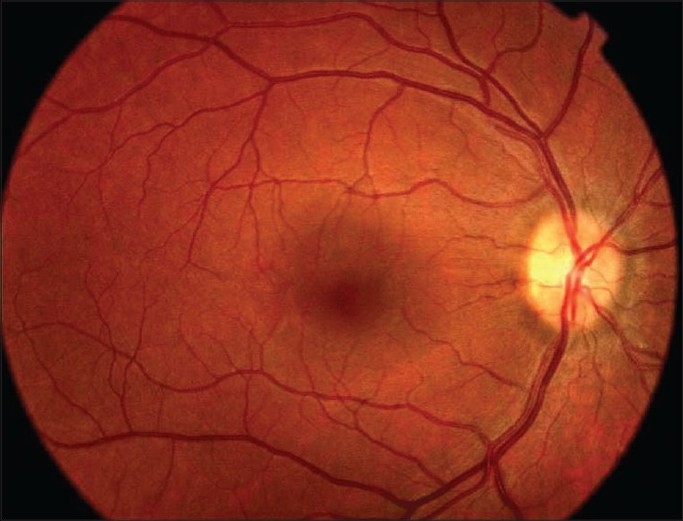
Color photo of the right eye one month after treatment. Note the resorption of the premacular hemorrhage and normal looking optic disc

A 28-year-old white female with a history of acute rheumatic fever and aortic valve insufficiency presented with poor physical condition associated with headache, fever, sweating and neck stiffness. She also showed sudden onset of painless blurred vision in the right eye associated with diplopia. Best corrected visual acuity of the right eye was 20/400 and the left eye was 20/50. She had right afferent pupillary defect and sluggish response to light on the left side. She had 30-45 prism diopters of right esotropia. The patient had reduced sensitivity to color vision higher in the red-green direction than in the blue-yellow direction in both eyes. The right fundus revealed 1^½^ disc diameter of premacular hemorrhage [Fig. 1]. Right and left optic disc showed marked swelling and hyperemia associated with peripapillary vascular changes and flame-shaped hemorrhages [Figs. 1 and 2]. Cranial CT and MRI scans were normal. Echocardiography disclosed vegetation on the aortic valve. Seroagglutination test for brucella was positive over 1/320 dilution. Cerebrospinal fluid (CSF) agglutination titer for brucella was positive over 1/32 dilution. CSF opening pressure was 150 mm H_2_O. She had normal CSF biochemistry without pleocytosis. Blood and CSF cultures for brucella were negative. She was diagnosed as having neurobrucellosis with right abducent nerve and bilateral optic nerve involvement, and endocarditis complicated by right premacular hemorrhage. She underwent removal of the vegetation and aortic valve replacement surgery followed by medical treatment with rifampicin 600 mg/day and doxycycline 200 mg/day for six months. Premacular hemorrhage, bilateral papillitis and right abducent nerve palsy regressed within a month of treatment [Fig. 3]. Visual acuity in both eyes returned to 20/20 after treatment. Color vision in both eyes also returned to normal.

## Discussion

High index of clinical suspicion coupled with seroagglutination tests have been recommended for diagnosis of brucellosis in endemic areas.[1] The most adequate cut-off point for seroagglutination has been considered as 1/160 with a sensitivity of 93% and specificity of 97%.[1] Seroagglutination test for brucella was positive over 1/320 dilution in our case. The incidence of neurobrucellosis has been reported to be high in endemic areas.[3] Detection of any titers of antibodies in CSF has been considered to provide evidence of neurobrucellosis.[3,4] However, CSF titer higher than 1/160 associated with increased CSF opening pressure which is greater than 200 mm H_2_O, pleocytosis, elevated protein, and reduced glucose concentrations have been considered for the diagnosis of brucella meningitis.[5] In addition, abnormal cranial CT and MRI findings have been reported for cases with brucella meningitis.[5] Our case showed CSF agglutination titers for brucella over 1/32 dilution. She had an opening pressure of 150 mm H_2_O associated with normal CSF biochemistry without pleocytosis. Her cranial CT and MRI scans were normal. She had mild meningeal signs associated with right esotropia and bilateral papillitis.

She was diagnosed as having neurobrucellosis with right abducent nerve and bilateral optic nerve involvement. Cardiac involvement has been considered to be rare but potentially lethal complication of brucellosis.[6,7] Brucella endocarditis is repoted to be mostly associated with acute rheumatic fever involving the aortic valve.[6] Past medical history of our patient was significant for acute rheumatic fever and aortic valve insufficiency. Echocardiography disclosed vegetation on the aortic valve. Premacular hemorrhage has been reported as a complication of subacute bacterial endocarditis.[7] Premacular hemorrhage detected in the right eye of our patient was considered to be related to an embolic process from the vegetation on the aortic valve. Cranial nerve involvement in neurobrucellosis has been reported previously,[8] however, neurobrucellosis and endocarditis complicated by premacular hemorrhage in our case from an endemic region was considered as a rare condition. Our patient showed dramatic clinical improvement after surgical removal of vegetation and replacement of aortic valve followed by specific treatment with rifampin and doxycycline for brucella. In summary, brucellosis should be considered in the differential diagnosis of optic neuritis, gaze palsy and endocarditis complicated by subhyaloid/preretinal hemorrhage in endemic areas.
